# Development of Natural-Based Bone Cement for a Controlled Doxorubicin-Drug Release

**DOI:** 10.3389/fbioe.2020.00754

**Published:** 2020-07-09

**Authors:** Rebecca Marie Dewhurst, Annachiara Scalzone, Joseph Buckley, Clara Mattu, Kenneth S. Rankin, Piergiorgio Gentile, Ana Marina Ferreira

**Affiliations:** ^1^Translational and Clinical Research Institute, Newcastle University, Newcastle upon Tyne, United Kingdom; ^2^School of Engineering, Newcastle University, Newcastle upon Tyne, United Kingdom; ^3^Department of Mechanical and Aerospace, Politecnico di Torino, Turin, Italy

**Keywords:** bone cement, nanoparticles, osteosarcoma, stem cells, regenerative medicine

## Abstract

Osteosarcoma (OS) accounts for 60% of all global bone cancer diagnoses. Intravenous administration of Doxorubicin Hydrochloride (DOXO) is the current form of OS treatment, however, systemic delivery has been linked to the onset of DOXO induced cardiomyopathy. Biomaterials including calcium phosphate cements (CPCs) and nanoparticles (NPs) have been tested as localized drug delivery scaffolds for OS cells. However, the tumor microenvironment is critical in cancer progression, with mesenchymal stem cells (MSCs) thought to promote OS metastasis and drug resistance. The extent of MSC assisted survival of OS cells in response to DOXO delivered by CPCs is unknown. In this study, we aimed at investigating the effect of DOXO release from a new formulation of calcium phosphate-based bone cement on the viability of OS cells cocultured with hMSC in vitro. NPs made of PLGA were loaded with DOXO and incorporated in the formulated bone cement to achieve local drug release. The inclusion of PLGA-DOXO NPs into CPCs was also proven to increase the levels of cytotoxicity of U2OS cells in mono- and coculture after 24 and 72 h. Our results demonstrate that a more effective localized DOXO delivery can be achieved via the use of CPCs loaded with PLGA-DOXO NPs compared to CPCs loaded with DOXO, by an observed reduction in metabolic activity of U2OS cells in indirect coculture with hMSCs. The presence of hMSCs offer a degree of DOXO resistance in U2OS cells cultured on PLGA-DOXO NP bone cements. The consideration of the tumor microenvironment via the indirect inclusion of hMSCs in this study can act as a starting point for future direct coculture and *in vivo* investigations.

## Introduction

Osteosarcoma (OS) is one of the main types of malignant skeletal tumors, accounting for 60% of diagnosed bone cancers globally (Ottaviani and Jaffe, 2009). In England, OS accounted for 34% of all diagnosed malignant bone sarcoma cases between the years 1979 and 2007 (Whelan et al., 2012). Most cases diagnosed were in one of two demographic groups; the first group was predominantly between 10 and 24 years old, while the second was 70 plus (Whelan et al., 2012). The common OS diagnosis is via plain radiographic imaging of the affected bone (Gorlick and Khanna, 2010) with around 70% of cases treated with chemotherapy and surgery (Collins et al., 2013). Along with advancements in the surgical technique, the addition of chemotherapy has dramatically increased the chances of survival. Current OS treatment involves surgical tumor removal combined with intravenous neo-adjuvant and adjuvant chemotherapy offering event free survival rates of 70% for localized cases and 20% for metastatic patients (Kansara and Thomas, 2007; Harrison et al., 2018). However, the current systemic administration of DOXO has been proven to induce side effects such as cardiomyopathy (Kumar et al., 2012; Abdullah et al., 2019), therefore localized delivery would be beneficial. The source of OS is still unknown, however, literature studies suggest it may stem from increased bone rates of growth during puberty (Rankin et al., 2012; Endicott et al., 2017) or the inhibition of tumor suppressor genes like *p53* (Kansara and Thomas, 2007). Mesenchymal stem cells (MSCs) have been linked to the survival of OS cells during chemotherapy by mediating proliferation, enabling metastasis and promoting drug resistance (Zheng et al., 2018). Two major forms of MSC are present in multiple types of cancer including OS patients, normal healthy MSCs (NMSC) and tumor MSCs (TMSC) (De Wever et al., 2014; Zheng et al., 2018). Broadly, NMSCs follow strictly regulated patterns of proliferation (Sun et al., 2014); however, after recruitment to tumor sites they undergo “educational” processes becoming TMSCs thus promoting cancer cell survival (Sun et al., 2014; Zheng et al., 2018). A major part of this educational process is the acquisition of a tumor-associated fibroblast (TAF) phenotype, allowing for the promotion of tumor cell growth (De Wever et al., 2014). This transformational process poses major implications in successful cancer treatment especially in OS patients due to the close proximity of NMSCs to diseased tissues.

The generation of materials and structures with promotional bone regenerative properties, growth, and controlled localized chemotherapeutic drug release preventing recurrence *in situ* can be beneficial following OS tumor removal. Natural polymers such as collagen and chitosan (CH) have been used to create osteoconductive substrates (Hockin and Simon, 2005; Zhang et al., 2014) when partnered with an apatite matrix, which provides the mechanical properties (Wang and Min-Hsiung, 2003). Moreover, CH has been used in wound healing with antibacterial properties (Islam et al., 2020), drug delivery carrier (Lee et al., 2009; Ashan et al., 2018), and bone regeneration (Saravanan et al., 2016). Ceramics including calcium phosphates, bioactive glasses and SiO2- and TiO2-(Hertz and Bruce, 2007; Mohammadi et al., 2011) have been used in the biomedical industry since the 1920s, due to their biocompatibility and good mechanical properties. They are most commonly used as the bioactive, reinforcing phase in bioactive tissue substitutes (LeGeros and LeGeros, 2003). Hydroxyapatite (HA) is one of the most commonly used calcium phosphates as a replacement bone apatite. Nano-hydroxyapatite (NHA), a synthetic calcium phosphate, offers a similar chemical composition, trace elements and crystal structure as native hydroxyapatite (Akram et al., 2014). As up to 70% of human bone tissue is HA (Dutta et al., 2015), calcium phosphates and synthetic HA are good candidates for bone tissue engineering applications. Calcium phosphate cements (CPC) and calcium sulphate cements (CSC) have both demonstrated to improve biocompatibility, bioactivity and osteoconductivity compared to HA alone under neutral pH conditions (Xu et al., 2017).

In a clinical context, CPCs have been developed and reported in several studies for the treatment of bone defects as injectable alternative fillers, supplementing internal fixation, during surgical treatment of bone metastasis, following tumor removal. CPC presence could help to avoid the spreading of tumor cells, which may result in new lesions developing in surrounding tissues, during an intralesional resection (Tanzawa et al., 2011). CPC has been exploited in several clinical applications, including traumatic and craniofacial surgery, spinal reconstruction and arthroplasty, showing promising results (Matsumanie et al., 2006). Relevant advantages of CPC are: (i) they are easily shaped to meet the bone defect’s dimensions; (ii) they have good osteoconductivity and biocompatibility (Frankenburg et al., 1998); (iii) they have chemical stability, enabling its loading with several drugs; (iv) and display a remarkable sustained-release capacity. Broadly, CPCs are replaced with newly formed bone once degraded, showing good levels of biodegradability and bio-reabsorbility (Weitao et al., 2008; He et al., 2018). Furthermore, loading anticancer drugs within the CPCs could offer the potential for reducing the risk of local tumor recurrence. Studies on CPCs used as localized drug delivery systems, loaded with synthetic polymer nanoparticles containing painkillers, antibiotics, and chemotherapeutic drugs (including OS treating drugs) to treat bone cancers demonstrated a reduced risk of local cancer recurrence due to localized and targeted delivery of drugs (Lopez-Heredia et al., 2011; Tanzawa et al., 2011; Farbod et al., 2016; Liang et al., 2020).

Recently, nanoparticles (NPs) have been widely used as drug delivery vehicles for multiple cancers, allowing for improvements in drug potency and targeted delivery (Pathak and Pathak, 2019). Different materials have been used to produce NPs, including metals such as iron (Singh et al., 2018), silver (Chaloupka et al., 2010) or gold (Patra et al., 2010) and biodegradable polymers such as lipid polymer base (Dehaini et al., 2016; Yalcin et al., 2020) and poly(D,L-lactide-co-glycolide) (PLGA) (Sokol et al., 2019); the latter enable a quick release of the drug carrier in cancer treatment. Doxorubicin Hydrochloride (DOXO), an anthracycline antibiotic used as a drug to treat multiple forms of cancers, has proven effective in stimulating apoptosis of lymphoma, leukemia and endothelium cells (Shafei et al., 2017; Mattu et al., 2018). It is also capable of inducing senescence in solid tumor depending upon concentrations administered (Kozhukharova et al., 2018). In OS treatment, DOXO is administered intravenously along with other chemotherapeutic drugs including methotrexate (MAP) and cisplatinum (Yu et al., 2019). *In vitro* studies assessing the effect of DOXO in human OS cell lines showed that cellular metabolic activity and growth of U2OS spheroids decrease after 24 h DOXO drug exposure, following endocytosis (Baek et al., 2016). However, U2OS cells are capable of developing DOXO resistance when overexposed to the drug (Buondonno et al., 2019). The use of NPs has shown promise in attempts to overcome associated limitations of free intravenous DOXO in cancer treatments such as breast cancer (Prados et al., 2012) and hepatic cancers (Krishnamurthy et al., 2017), indicating DOXO loaded NPs could be a viable method of stimulating residual osteosarcoma cell apoptosis. Attempts incorporating therapeutic NPs loaded with Adriamycin (ADM, the commercial name for DOXO) into porous NHA and collagen scaffolds have shown successful growth inhibition of human osteosarcoma cell line MG63 (Rong et al., 2016). From this work, it was demonstrated that the combination of NHA and collagen scaffolds loaded with DOXO NPs can be beneficial in inhibiting tumor growth while assisting bone regeneration (Rong et al., 2016). This success has also been reflected in *in vivo* studies conducted in rats (Hoving et al., 2005) and murine models of Saos2 and MG63 (Ma et al., 2015), showing the immense benefit of DOXO loaded biomaterials.

In this study, we aimed at investigating the effect of DOXO release from biodegradable/bioactive constructs on the viability of OS cells cocultured with hMSC *in vitro*. For this purpose, a new formulation of biodegradable calcium phosphate-based bone cement with radiopaque properties was developed. NPs made of PLGA were loaded with DOXO and incorporated in the formulated bone cement to achieve local drug release. The impact of DOXO release from bone cement on cell viability and metabolic activity of U2OS osteosarcoma cells either in monoculture and in coculture with hMSCs was assessed in vitro. This pioneering study shows that hMSCs play a modulatory role on U2OS cells survival when exposed to DOXO.

## Materials and Methods

### Materials

For bone cement preparation chitosan (CH, 95% deacetylation, molecular weight 500 kDa) was provided by Heppe Medical Chitosan. Gelatin Type A (G, derived from pig skin, CAS No. 9000-70-80), Nano-Hydroxyapatite (NHA, particle size between 49 and 152 nm, molecular weight 502.31, CAS No. 12167-74-7), Bismuth Salicylate (BS, molecular weight of 362.09 g/mol, CAS No. 14882-18-9) Acetic Acid (Glacial, Reagent Plus, ≥99%, molecular weight 60.05, CAS No. 64-19-7), and Sodium Hydroxide (NaOH, Ultra dry crystals, 99.99% trace metals basis, molecular weight 40.00, CAS No. 1310-732), where all purchased from Sigma-Aldrich. Genipin (GP, CAS No. 6902-77-8) was supplied from Challenge Bioproduct Co. For nanoparticle preparations Resomer^®^ RG 858 S (ester terminated Poly(D,L-lactide-co-glycolide (PLGA), lactide:glycolide 85:15, molecular weight 170,000–230,000 Da, CAS No 26780-50-7), Acetone (≥99.9%, molecular weight 58.08, CAS No. 67-64-1), Tween 80 (CAS No. 9005-65-6) and Dimethyl Sulfate (DMSO, molecular weight 78.13, CAS No. 6768-5) were all purchased from Sigma Aldrich. For cell culture tests, Fetal Bovine Serum (FBS, ThermoFisher, Gibco CAS No. 10500056), penicillin/streptomycin (P/S, Sigma-Aldrich, P0781100 mL), Human Fibroblast Growth Factor (hFGF-2, Sigma-Aldrich, 106096-939), L-Glutamine (LG, 5 mM, Sigma-Aldrich, TMS-002), Phosphate Buffered Saline (PBS Sigma-Aldrich, MFCD00131855), Trypsin/EDTA (Sigma-Aldrich, MFCD00130286), Dulbecco’s Modified Eagles Medium (DMEM, Sigma-Aldrich, Gibco high glucose 4500 mg/L, 11995-065) were used. Doxorubicin Hydrochloride (DOXO, European Pharmacopoeia Reference Standard, molecular weight 579.98, CAS No. 25316-40-9) was purchased from Sigma-Aldrich.

### Bone Cement Characterizations

#### Bone Cement Preparation

Four different bone cement formulas were initially tested (40/60, 50/50, 60/40, and 70/30 of polymer to NHA). Briefly, a 2:1 weight/weight (w/w) solution of CH:G was dissolved into 2% Acetic Acid and left under magnetic stirring at 40°C until dissolved (Mi, 2005; Pullieri et al., 2008). Then, NHA powder was added to polymeric solutions, forming cements with 40/60, 50/50, 60/40, and 70/30 ratios of CH:G to NHA, followed by BS addition (quantities shown in Table 1). Once mixed, the pH of the solution was adjusted to 6 by adding drop wise 1M NaOH, then the mixture was stirred for 1 h at 40°C until NHA was homogenously dispersed. The cross-linker GP was added to the mixture and stirred vigorously until homogenous (about 10 min). Cements were then transferred to 24 well plates and incubated for 24 h at 37°C for solidification. Once GP reacted, a dark blue color was observed and cements were stored at 4°C for further analysis.

**TABLE 1:** Percentages in weight (%wt) of the different components for bone cement preparation at 40:60, 50:50, 60:40, and 70:30 CH+G:NHA ratios (contains Nanohydroxyapatite (NHA), Bismuth Salicylate (BS) as radiopaque agent, Genipin (GP) as crosslinker, chitosan (CH) and gelatin (G) as biopolymers).

Component	Composition of Bone Cement (CH+G to NHA)			
	40/60	50/50	60/40	70/30
Chitosan (CH)	26.7% wt	33.3% wt	40% wt	46.67% wt
Gelatin (G)	13.3% wt	17.7% wt	20% wt	23.33% wt
Nanohydroxyapatite (NHA)	60% wt	50% wt	40% wt	30% wt
Bismuth Salicylate (BS)	15% w/w	15% w/w	15% w/w	15% w/w
Genipin (GP)	7.5% w/w	7.5% w/w	7.5% w/w	7.5% w/w

#### Curing Time and Injectability

To investigate both injectability and curing time of four bone cements compositions, 5 ml of each of bone composition cements was loaded in a 5 ml syringe, following mixing step. The injectability was calculated as the percentage of mass that can be extruded from the syringe during about 1 min, as described elsewhere (Xiaopeng et al., 2008). For this, the mass of the syringe loaded and unloaded with the bone cement was recorded. This process was repeated three times per each cement composition.

To investigate cement curing time, 2 mL of bone cements containing GP were transferred in triplicate into clear plastic vials and incubated at 37°C. The setting time was determined by tilting each vial at least 45° angle every 60-s intervals until set. Cements were set once they stopped flowing by tilting the vial, showing firm consistency and color change from white to blue as evidence of GP cross-linking reaction.

#### Unconfined Compression Test

The compressive mechanical properties of four bone cements compositions (without DOXO or PLGA-DOXO NPs) specimens was measured by using a Universal testing machine (SHIMADZU ES-SX, Japan) equipped with 50N loading cell at a crosshead speed of 1 mm min^–1^. Test specimens were cylinder-shaped cements with a 1.3 cm diameter and an average height of about 1 cm. A pre-load of 0.15N was applied, and further sample loading was recorded until the specimen was compressed to around 80% of its original height before break. Compression tests of five samples for each composition was evaluated at room temperature in dry state, stress was calculated by dividing the applied force with the initial scaffold surface area, whereas strain was calculated from the displacement of the scaffolds in relation to the original thickness. Young’s modulus (E) was also calculated as the slope of the linear elastic regime (0–15%).

#### Spectroscopy Fourier Transform Infrared (FTIR) – Attenuated Total Reflectance (ATR)

FTIR spectra were obtained using a Spectrum Two PE instrument equipped with attenuated total reflectance (ATR) crystal (diamond crystal) (Frontier PerkinElmer Inc., United States). Bone cements were frozen at −0°C for 48 h and then freeze dried for 48 h (Alpha 1-2 LD plus, CHRIST, Germany). Samples were placed directly onto the ATR crystal and spectra were collected in transmittance mode. FTIR spectra measurements were an average of 36 scans at 4 cm^–1^ resolution in the wavelength range of 4000 - 550 cm^–1^. Triplicate samples of each bone cement composition (without DOXO or PLGA-DOXO NPs) were analyzed.

#### Radiopacity Properties

The four bone cements (40:60, 50:50, 60:40, and 70:30 ratios of CH+G:NHA) were investigated by X-ray to confirm if BS concentration offers viable radiopacity. Triplicate samples of each cement containing 15% w/w BS were imaged via X-ray at 40 kV, with two exposure times of 11 mAs and 5.6 mAs (Royal Victoria Infirmary, Newcastle). Radiopacity was also investigated via phantom imaging (Radiology Department, RVI Hospital Newcastle) used as control, to compare the radiopacity of the samples to the actual radiopacity of bone. Bone cements visibly brighter than phantom were considered to have a viable radiopacity.

#### Pore Size and Degradation

Mass loss was investigated for 40/60 bone cement (in absence of DOXO or PLGA-DOXO NPs). The degradation degree of samples (*n* = 3, with dimensions of 1.3 cm diameter and height 1 cm) was calculated gravimetrically using an analytical balance (Sartorius, Sartorius AG). Briefly, freeze dried samples were initially weighted (day 0) and then immersed into 5 mL of Phosphate Buffered Solution (PBS) (Sigma Aldrich, United Kingdom) at pH 7.0, with the solution refreshed weekly for 14 days. Following 14 days of incubation at 37°C, samples were removed from the buffer solution and freeze dried for re-weighing of sample mass.

The inner morphology of freeze-dried (40/60) bone cement, with and without free DOXO (40 μM) and PLGA-DOXO NPs (100 μM), were analyzed by Scanning Electron Microscopy (SEM, Hitachi TM3030 Tabletop, Germany) at accelerating voltage of 10 kV. The samples were cut into small squares and fixed on the aluminum stub using carbon tape. SEM images were analyzed using an image software (ImageJ) for pores size measurement. Three images per type of sample were analyzed measuring at least 30 pores for each one. The pore size was averaged, assuming all pores were circular.

Changes of pH of bone cements (40/60) in absence (0 μM) presence of PLGA-DOXO NPs (40 μM and 100 μM) at physiological conditions (PBS pH 7.0, 37°C) were monitored over 3 days by using portable FG2-Kit Five Go^TM^ pH meter (Mettler Toledo Ltd., United Kingdom).

#### Drug Release

Bone cements (40/60 composition) containing free DOXO (to achieve a final concentration of 40 μM and 100 μM in the tissue culture well) and PLGA-DOXO NPs (to achieve the same final DOXO concentration of 40 μM and 100 μM in tissue culture well) were prepared in triplicate in a 48 well plate, as described previously. DOXO drug release was determined by immersing samples in 5 mL of a PBS solution (pH 7.0) and incubating at 37°C over 7 days’ time-period. At pre-determined time intervals (1, 2, 3, 4, and 7 days), samples solutions were removed and replaced with fresh buffer. Samples solutions were transferred to a flat-bottom 96 well plate for further UV–Vis spectroscopy analysis in absorbance at 480 nm, and compared to a standard curve generated using free DOXO in PBS at known concentrations. All tests were performed in triplicate and results were expressed as cumulative released DOXO (% of the drug loaded in the construct), compared to a standard curve generated using free DOXO in PBS at known concentrations.

### PLGA-DOXO NP Characterizations

#### PLGA-DOXO NP Preparation

DOXO-loaded nanoparticles were prepared by the nanoprecipitation method, by adapting the original method described elsewhere (Mattu et al., 2012). Briefly, 30 mg of PLGA ester-terminated was dissolved into 3 mL pure acetone, and 100 μL of 10 mM DOXO stock solution (1:1 of DMSO:PBS) was added to the polymer solution (2% w/w with respect to the polymer weight). PLGA-loaded NPs were prepared by adding dropwise this primary solution into 7 ml of 1% w/v deionized water containing Tween 80 (10 mg/mL), followed by gently stirring for 1 h at room temperature. PLGA-DOXO NPS were collected by selective centrifugation steps at 3.000 rpm, followed by centrifugation at 10.000 rpm. The nanoparticles pellet obtained from the two centrifugation steps were collected and washed three times with distilled water. The final particle suspension was frozen at −20°C and subsequently freeze-dried for 48 h (Alpha 1-2 LD plus, CHRIST, Germany).

#### Encapsulation Efficiency of Doxorubicin Into PLGA Nanoparticles

Encapsulation efficiency (EE) was determined via UV/VIS spectroscopy. Freeze dried PLGA-DOXO NPs were incubated in PBS (1 mg/ml) for 30 min at 37°C, centrifuged at 1500 rpm for 15 min, and the supernatant was collected. This process was repeated by suspending remaining particles in PBS (by vigorously shaking for 5 min) and centrifuging at 1500 rpm. Supernatants collected from each step were analyzed at 480 nm to detect DOXO. Each sample was read in triplicate and, referred to a standard curve generated using free DOXO in PBS at known concentrations. The EE of PLGA-DOXO NP was calculated using the following equation;

$$\begin{array}{c} E\,n\,c\,a\,p\,s\,u\,l\,a\,t\,i\,o\,n\\ Efficiency\left(\%\right)= \end{array}\,\left(\frac{\begin{array}{c} \left[d\,r\,u\,g\,r\,e\,l\,e\,a\,s\,e\,d\,i\,n\,P\,B\,S\right]+\\ \left[d\,r\,u\,g\,f\,r\,o\,m\,P\,B\,S\,w\,a\,s\,h\right] \end{array}}{\begin{array}{c} {}[drugaddedduring\\ NPformation] \end{array}}\right)\times 100$$

#### Morphology and Size of Polymeric NP

The morphology of freeze-dried PLGA-DOXO NPs was analyzed by Scanning Electron Microscopy (SEM, Hitachi TM3030 Tabletop, Germany) at accelerating voltage of 10 kV. SEM images were obtained at a magnification of X 6K and analyzed using an image software (ImageJ) for size measurement. Moreover, the particle size analysis and size distribution analysis were performed by using a dynamic laser light scattering technology (Malvern Zetasizer, Nano ZS) at room temperature in ultrapure water. PLGA-DOXO NPs were suspended in DI water (0.5 mg/mL) with a material refractive index of 1.49 and absorbance of 0.001 to obtain NP hydrodynamic diameter (nm). Results are an average of triplicate samples.

### Cellular Characterizations

#### Cell Culture

Bone marrow derived hMSCs (Sigma-Aldrich, SCC034) at passage 1 were cultured in Dulbecco’s Modified Eagles Medium (DMEM) supplemented with 10% Fetal Bovine Serum (FBS), 1% penicillin/streptomycin (P/S) and 8 ng/mL Human Fibroblast Growth Factor (hFGF) at 37°C until 70% confluent. Human U2OS cells (Sigma-Aldrich, 92022711) at passage 21 were cultured in DMEM supplemented with 10% FBS, 1% P/S and 1% L-Glutamine at 37°C until 80% confluent. Cells were washed with PBS before trypsinization with Trypsin/EDTA, centrifugation and resuspension in their respective media. Media was changed every 2–3 days. For spheroids, 2 × 10^5^ U2OS cells were seeded in a “v” bottom 96 well plate and left for 3–5 at 37°C, 5% CO_2_, 90% humidity. Media was changed every 2–3 days.

#### IC50 Characterization and DOXO Concentration Selection for Cement Loading

For IC50 measurement, U2OS were cultured in a 48 well-plate and in 96 well “V” bottom plate for U2OS spheroids formation at 2 × 10^5^cells/well, while hMSCs were cultured in a 48 well-plate at 2 × 10^4^ cells/well with 24 h incubation for cell attachment. Then, media was removed and replaced with media suspension containing DOXO at concentrations ranging from 0 μM (no DOXO) to 200 μM for investigating the effect of different drug doses. Following 24 h incubation, drug media suspension was removed, and cell viability was assessed via the MTT assay in absorbance at λ = 570 nm, as described below in metabolic activity section. Then, IC50, which indicates the half maximal effective drug concentration that induces half cellular response between the baseline and the maximum response at specific exposure time, was calculated by the non-linear regression (sigmoidal dose–response) using GraphPad (GraphPad Prism Software Inc., San Diego, CA, United States). From U2OS IC50 obtained results, DOXO concentrations of 40 μM and 100 μM were selected to be incorporated into bone cements (40/60), either as free DOXO or loaded into NPs. The cellular behavior of U2OS mono- and indirect cocultures (U2OS and hMSCs) in presence of bone cements containing 40 μM and 100 μM free DOXO or/and drug-loaded into NPs was investigated. All tests were performed in triplicate.

#### Cellular Seeding Onto Bone Cements

From physical-chemical characterizations, the 40/60 composition was selected for further bone cement cytocompatibility assessment. Bone cements with and without free DOXO and PLGA-DOXO NPs (40 μM and 100 μM DOXO) were prepared and sterilized by UV light (245 nm) prior to cell seeding. Samples were then incubated for 30 min at 37°C in U2OS cell media, while hanging millipore cell inserts (pore size 8.0 μm) were incubated in hMSC media. Following this, U2OS media was read in absorbance at 480 nm (using the FLUOstar OMEGA microplate reader) to determine any potential DOXO drug release during pre-incubation. U2OS cells (1 × 10^5^) were seeded directly onto cements placed in well bottom, while hMSCs (2 × 10^4^) were seeded onto hanging inserts. Cells were left at 37°C for 15–20 min to allow their adhesion, prior to the well topping up with the media. For immunostaining analysis, sudan black solution was used onto bone cement prior to cell seeding to minimize background fluorescence given by Genipin. All tests were performed in triplicate.

#### Metabolic Activity

Metabolic activity was assessed via the MTT assay (Sigma-Aldrich, ≥97.5%, HPLC, molecular weight 414.32, CAS No. 298-93-1). Briefly, 2 × 10^5^ U2OS cells were seeded directly onto (40/60) bone cements with free DOXO and PLGA-DOXO NPs at concentrations 40 μM and 100 μM, and cements without free DOXO or PLGA-DOXO NPs (0 μM). In co-culture systems, hMSCs were seeded onto Millipore cell inserts (pore size 8.0 μm) at 2 × 10^4^ cells/insert, jointly with U2OS cultured onto bone cements. The metabolic activity was measured individually for U2OS and hMSCs (if in co-culture) by transferring the inserts with hMSCs into a new 24 multiwell plate. Following cellular incubation 24 and 72 h, the culture media was removed and 200 μL of 1 mg/mL MTT in DMEM (phenol red and serum free) was added to each well. Samples treated with MTT reagent were incubated for 2 h at 37°C, protected from light. Media was then carefully removed and 200 μL of DMSO was added to each well to solubilize the tretrazolium crystals. The multiwell plate was covered with tinfoil and agitated on an orbital shaker for 20 min. Solubilized formazan (100 μL) was transferred to a 96-multiwell plate and read at λ = 570 nm using the FLUOstar OMEGA microplate reader. Standard curves for hMSCs, U2OS, and U2OS spheroids were generated at known cell densities. All tests were performed in triplicate.

#### Cytotoxicity by LDH and Live/Dead Assays

Cytotoxicity of U2OS cells in mono- and co-culture (with hMSCs) grown on bone cements with and without PLGA-DOXO NPs was measured using the Lactate Dehydrogenase (LDH) Assay Kit (Fluorometric, ab197000, Abcam, United Kingdom) and Live/Dead assay (LIVE/DEAD^®^ Cell Imaging Kit, Life Technologies, United Kingdom). LDH kit was used according to manufacturer instructions. In this assay, LDH converts lactate into pyruvate and NADH, which reacts with the specific fluorescent probe to generate an intense fluorescent product (Ex/Em = 535/587 nm). Live/Dead Cell Imaging Kit was used according to the manufacturer’s instructions. This fluorescence-based kit uses calcein AM and ethidium bromide to identify live cells (green) from the dead cells (red) from cell populations. Samples were washed twice with PBS before incubation with staining. Briefly, 4 μM ethidium homodimer-1 and 10 μM calcein were diluted in DPBS, and samples were incubated with Live/Dead staining for 30 min at 37°C. Images were collected at 1 and 3 days using an EVOS^TM^ M5000 fluorescence microscope equipped.

#### Cellular Morphology by Confocal Immunostaining and SEM Analysis

For immunostaining analysis, after 72 h, samples were fixed in pre-warmed 4% w/v paraformaldehyde (PFA) for 30 min at room temperature (RT) prior to cell permeabilization using 0.1% v/v Tween20^®^ in PBS (PBS/Tween20^®^). For the cement, DAPI solution (Vector Laboratories, United Kingdom) (1:2500 in 0.1% PBS/Tween20^®^) was added to the samples for 10 min at RT and then washed three times with 0.1% PBS/Tween20^®^. For the insert, DAPI staining was performed in the same way as for the cement and after that Phalloidin staining was carried out. Rhodamine-phalloidin was prepared using phalloidin-tetramethylrhodamine B isothiocyanate (1:1000 in 0.1% PBS/Tween20^®^) for 20 min at RT. Then, samples were washed with 0.1% PBS/Tween20^®^ solution. Experiments were light sensitive, and images were collected at 72 h using a Nikon A1R inverted confocal microscope. For the SEM analysis, at 72 h, samples were fixed in pre-warmed 2% Glutaraldehyde overnight, rinsed in PBS twice and dehydrated in ethanol grades: 30 min in each 25, 50, and 75% EtOH and 1 h in 100% EtOH (twice). Samples were stored at 4°C in 100% EtOH until critical point dried using a BAL-TEC 030 Critical Point Dryer (Leica Geosystems Ltd., Milton Keynes, United Kingdom). Finally, cements were mounted on carbon disks (TAAB Laboratory Equipment) and gold-coated using a Polaron E5000 SEM Coating unit (Quorum Technologies Ltd., United Kingdom). Samples were imaged with a Tescan Vega LMU scanning electron microscope. The brightness of images was adjusted to aid visibility of cell nuclei.

### Statistical Analysis

Statistical analysis was performed using GraphPad Prism 8.2.1 with three independent experiments performed. Data was analyzed using two-way ANOVA (Bonferroni, unless differently stated) and paired *t*-test, with the significant difference set at *p* < 0.05. Data is presented as mean ± SD.

## Results

### Bone Cement Physical-Chemical Characterizations

#### Curing Time and Injectability

**FIGURE 1:**
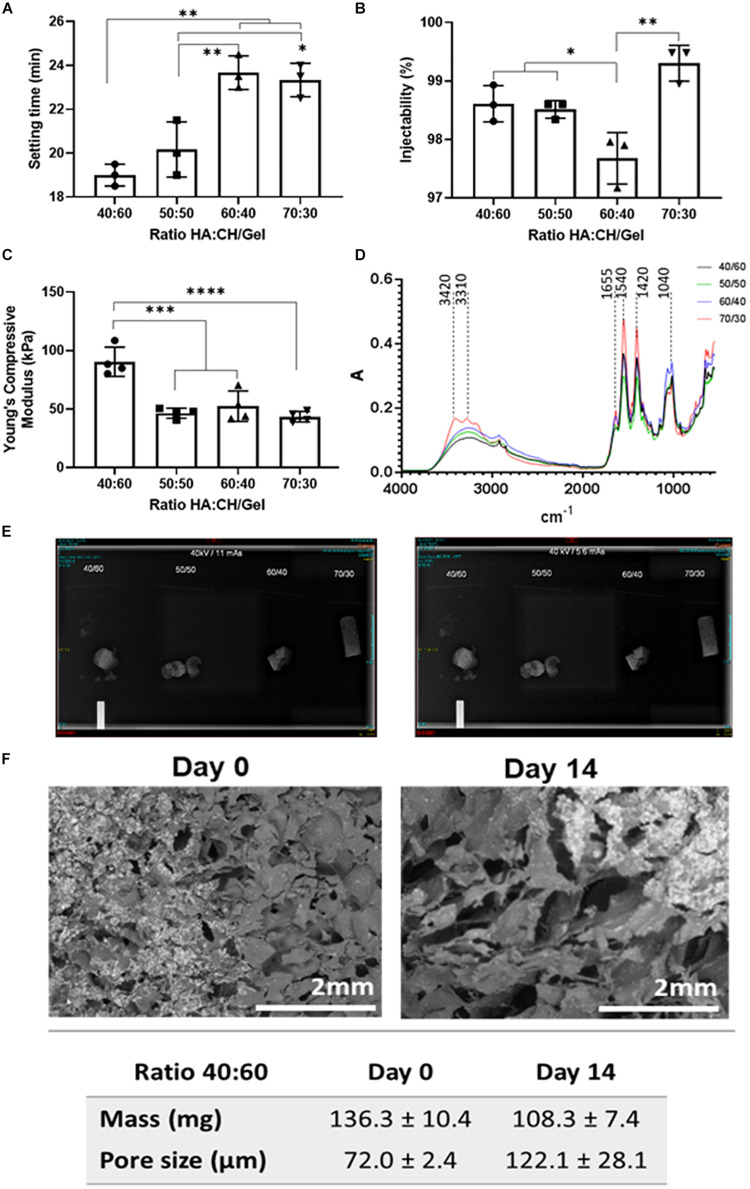
**(A)** Setting time (in minutes), **(B)** Injectability as (%), **(C)** Compressive Young modulus, **(D)** FTIR-ATR spectra, **(E)** 40 kV 11 mAs (left) and 5.6 mAs X-Ray (right) Images visible for bone cements at different ratios of hydroxyapatite and Chitosan/Gelatin 40/60, 50/50, 60/40, and 70/30. **(F)** SEM micrographs of bone cements (40/60) after 14 days incubation in PBS at 37°C. Statistics: *p < 0.05, **p < 0.007, ***p < 0.0007, ****p < 0.0001.

Setting time was investigated in all four compositions of cements, with the 40/60 ratio found to have the quickest mean setting time of 19 min (±0.5 min), with data presented in Figure 1A. As NHA content decreased, there was an increase in the setting time of the cement with significant differences when comparing cements with a 50/50 (20.17 ± 1.26 min), 60/40 (23.67 ± 0.76 min) and 70/30 (23.33 ± 0.76 min) ratio after statistical analysis. Injectability ranged between 97.23 and 99.6% in cements with varying concentrations of NHA and is presented in Figure 1B. Cements with 70/30 NHA compositions had the best injectability (99.30 ± 3.0%) followed by 40/60 (98.61% ± 0.31%), 50/50 (98.51 ± 0.15%) and 60/40 (97.67 ± 0.44%). Significant differences were found via one-way ANOVA (Bonferroni) with injectability decreasing with decreased levels of NHA, with an exception observed in the 70/30 cements.

#### Mechanical Properties: Compression Tests

The cements containing either equal or less NHA to CH and G were found to have no significant difference in Young’s compressive modulus (50/50: 48.71 ± 1.85 kPa, 60/40: 46.47 ± 6.45k Pa and 70/30: 45.06 ± 3.89 kPa, respectively), data presented in Figure 1C. However, when the ratio of CH and G to NHA was increased to 40/60 considerable increases in young’s compressive modulus were observed (97.04 ± 18.04 kPa). A significant difference was found between cements containing 40/60 CH and G to NHA and all other NHA concentrations.

#### Chemical Analysis by FTIR-ATR

Fourier Transformed Infrared analysis in Attenuated Total Reflectance spectra were measured for the four compositions of bone cement (40/60, 50/50, 60/40, and 70/30), with results shown in Figure 1D. In general, spectra of four bone cements showed Gelatin (G) peaks between 3300 and 3500 cm^–1^ for O-H and N-H bands, primary and secondary amide bands (C = O and N-H) appearing at 1655 cm^–1^ and 1540 cm^–1^, respectively. Similarly, for chitosan (CH), peaks are observed between 3400 and 3500 cm^–1^ due to vibrational stretching of N-H and O-H groups. Due to the content of amide bonds in both, chitosan and gelatin, there is an overlapping in C = O band at 1655 cm^–1^ and N-H peaks at 1540 cm^–1^ in bone cement spectra. Also, peaks at 1062–1150 cm^–1^ associated with C-O-C stretching and at 2970–2880 cm^–1^ stretching vibration of aliphatic groups (-CH_2_ and -CH_3_) are observed. On this regard, by increasing the content of the biopolymers (gelatin and chitosan), it was evidenced an increase of the peaks in the range of 3300–3500 cm^–1^ as well as those corresponding to the amide group (C = O bonds at 1655 cm^–1^ and the deformation of N-H groups at 1540 cm^–1^). While, an increasing of the NHA content in bone cements showed an increasing of two peaks intensity at 869 cm^–1^ (aliphatic P-O-C stretching) and 1041 cm^–1^ (characteristic of the PO_4_^2–^).

#### Radiopacity

Radiopacity properties of the four different compositions of bone cement (40/60, 50/50, 60/40, and 70/30) were assessed via X-ray imaging at 40 kV at 11 mAs and 5.6 mAs. The images obtained (Figure 1E) show that all bone cement compositions were visible, exhibiting radio contrast properties and bright white shapes. Furthermore, X-rays demonstrated that the cements containing BS were quite homogenous, except slight speckled effects observed due to potential uneven BS distribution.

#### Morphology and Structure by Quantification of Pore Size and Degradation

Initial bone cement formulation (40/60) was studied in terms of degradation and mass changes over 14 days period of incubation at physiological conditions. From Figure 1F, it can be observed that bone cement scaffolds have changed their morphology over time by increasing the size of the pores of about 50.1 μm (from day 0 to day 14) and significant mean mass reduction (about 28 mg) following 14 days incubation period, as consequence of materials degradation.

In Supplementary Figure S3A are reported the SEM images of bone cements (40/60 ratio), containing either no DOXO (0 μM), free DOXO (40 μM) and PLGA-DOXO NPs (100 μM), which were analyzed using ImageJ software to calculate the mean pore area shown in Supplementary Figure S3B. The morphology and pore size were found to drastically change with the addition of DOXO (*p* < 0.0001) for 40 μM and 100 μM compared to the control (0 μM). In particular, a significant reduction of the mean pore size is observed when DOXO is incorporated into bone cements. From SEM micrographs, it is observed macroscopically that the number of pores increase while the pore size is significantly reduced when DOXO (40 μM and 100 μM) is incorporated in the bone cements. Bone cements without DOXO had the largest pore area (0.56 μm^2^) compared to the 40 μM and 100 μM cements (0.0195 μm^2^ and 0.0198 μm^2^, respectively).

#### Drug Release of Doxorubicin

**FIGURE 2:**
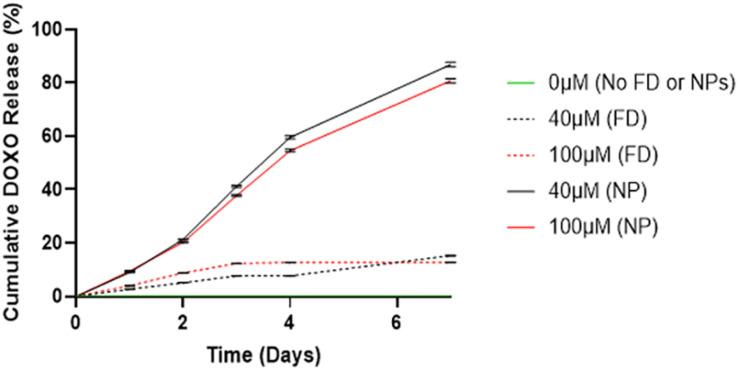
Drug release profiles of DOXO from bone cements (40/60) containing no DOXO (0 μM), free DOXO (40 μM and 100 μM) and PLGA-DOXO NPs (40 μM and 100 μM), into PBS after 7 days incubation at 37°C. Data presented as mean ± standard deviation, (*n* = 3 replicates).

In Figure 2, a significant difference is observed between DOXO release between cements (40/60) containing PLGA-DOXO NPs and free DOXO at concentrations of 40 μM and 10 μM (*p* < 0.0007 and *p* < 0.0146, respectively). A higher cumulative drug release (%) is detected in cements containing PLGA-DOXO NPs, with around 80% of DOXO released from cements at 40 μM and 100 μM DOXO over 7 days. However, less than 20% of DOXO was released from cements without NPs, reaching a plateau in drug release following 2–3 days of incubation.

### PLGA-DOXO NP Characterizations

#### Morphology, Size Measurements and Encapsulation Efficient of PLGA-DOXO Nanoparticles

**FIGURE 3:**
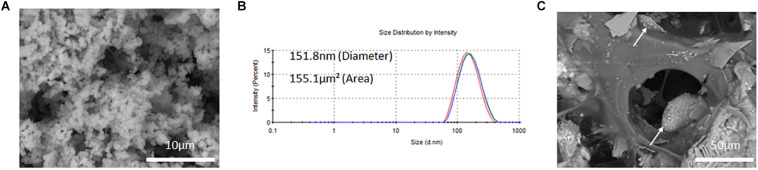
**(A)** SEM image of free PLGA-DOXO NPs. Scale bar represents 10 μm. **(B)** Zetasizer DLS readings of PLGA-DOXO NPs suspended in DI water. **(C)** SEM image of PLGA-DOXO NPs loaded bone cement (40/60 composition) (visible as white spheres). Scale bar represents 50 μm.

SEM micrographs of freeze-dried PLGA-DOXO NPs are shown in Figure 3A and evidence a spherical morphology, not affected by the freeze-drying process. The EE of DOXO was about 32.8 ± 0.6%. PLGA-DOXO NPs (*n* = 3) were found to be monodisperse in size with a mean hydrodynamic diameter of 152 nm (Figure 3B) and a polydispersity index of 0.181. The amount of NPs to be embedded in the cement required to achieve final DOXO concentrations of 40 μM (0.6 mg/ml) and 100 μM (1.5 mg/ml) was calculated based on the EE.

### Cellular Characterizations

#### Assessment of IC50 of Doxorubicin

**FIGURE 4:**
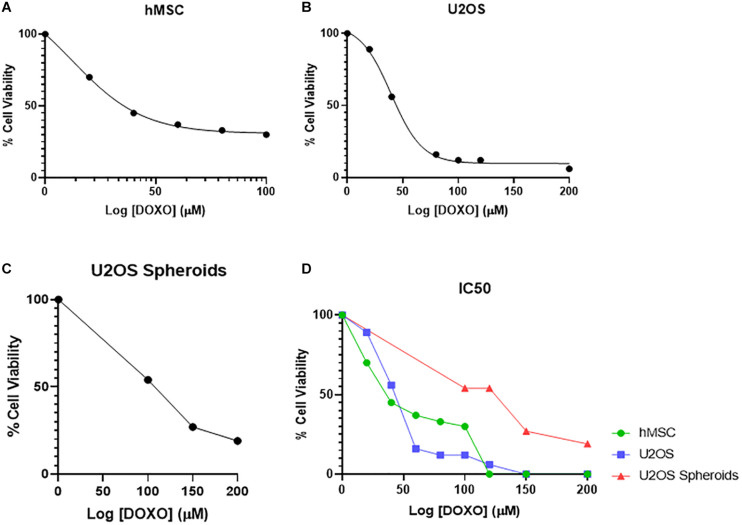
Mean percentage cell viability of **(A)** hMSC, **(B)** U2OS cells, and **(C)** U2OS spheroids after 24 h incubation with varying concentrations of free DOXO (0–200 μM) in the respective growth media at 37°C, used to identify the IC50 value of DOXO in these cell types (*n* = 3 replicas, ±SD) **(D)**.

The IC50 for U2OS and hMSCs cells at different DOXO concentrations are shown in Figures 4A,B as percentage of cell viability. Obtained IC50 values (min and max range for U2OS) were used identify the two DOXO concentrations to be used (40 μM and 100 μM), either as free DOXO loaded into NPs in bone cements. U2OS cells appear more responsive to DOXO when compared to hMSCs (27.359 μM and 31.147 μM, respectively). Further analysis on IC50 for U2OS spheroids (96.158 μM) was also explored to understand DOXO drug effect in micromass of tumoral cells (Figure 4C). In general, by contrasting IC50 (as % cell viability) and cell response to different DOXO concentrations (Figure 4D), U2OS cells appear more vulnerable against DOXO drug when compared to hMSCs and U2OS spheroid.

#### Osteosarcoma and hMSCs Metabolic Activity

**FIGURE 5:**
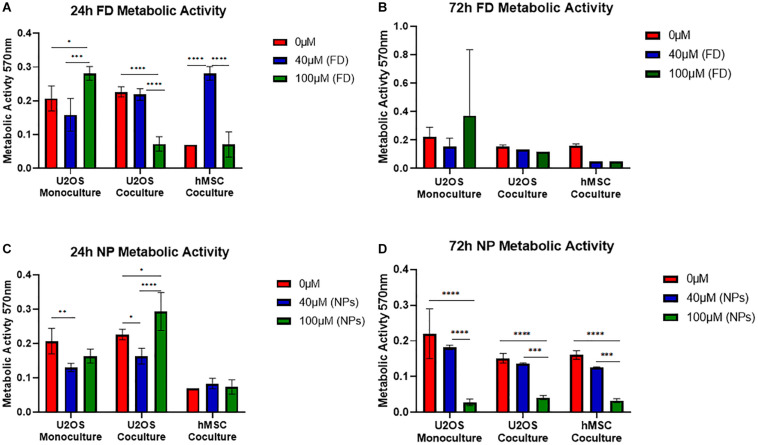
Mean metabolic activity of U2OS cells in monoculture, U2OS cells in coculture and hMSCs in coculture grown on bone cements (40/60 composition) without DOXO (0 μM) and with Free DOXO (FD, 40 μM and 100 μM) **(A)** 24 and **(B)** 72 h. Mean metabolic activity of U2OS cells in monoculture, U2OS cells in coculture and hMSCs in coculture grown on bone cements with a 40/60 CH+G to NHA composition on cements without PLGA-DOXO NPs (0 μM) and with PLGA-DOXO NPs (40 μM and 100 μM) after **(C)** 24 and **(D)** 72 h. (*n* = 3 replicas, *p < 0.05, **p < 0.01, ***p > 0.001, and ****p > 0.0001, respectively).

The metabolic activity of U2OS cells cultured either in monoculture or indirect coculture with hMSCs, onto bone cement (40/60) with free DOXO (FD) or PLGA-DOXO NPs (NPs), at 40 μM and 100 μM, or without FD or NPs (0 μM, referred as control) are shown in Figure 5. Figure 5A shows that there was a significant difference in the metabolic activity of monocultured U2OS cells after 24h between 0 and 100 μM (*p* < 0.0107) and 40 and 100 μM (*p* < 0.0001). This trend was also seen in U2OS cells cocultured with hMSCs (Figure 5A) between 0 and 100 μM (*p* < 0.0001) and 40 and 100 μM (*p* < 0.0001). Significant differences were also found in hMSC metabolic activity (Figure 5A) between 0 and 40 μM (*p* < 0.0001) and 40 and 100 μM (*p* < 0.0001). In comparison, no significant difference was found in the metabolic activity of U2OS cells in mono- or coculture with hMSCs after 72 h on any cement containing free DOXO (40 or 100 μM) (Figure 5B). A similar trend was reflected in hMSCs metabolic activity after 72 h (Figure 5B).

In regards to cultures grown on bone cement (40/60) loaded with PLGA-DOXO NPs significant differences were found between monocultured U2OS cells at 0 and 40 μM (*p* < 0.0064), and cocultured U2OS at 0 and 40 μM (*p* < 0.0243), 0 and 100 μM (*p* < 0.0176) and 40 and 100 μM (*p* < 0.0001) after 24 h (Figure 5C). There was no significant difference in hMSC metabolic activity after 24 h (Figure 5C). After 72 h of culture with cements loaded with PLGA-DOXO NPs (Figure 5D), a significant difference in metabolic activity was observed in mono-cultured U2OS between 0 and 100 μM (*p* < 0.0.0001) and 40 and 100 μM (*p* < 0.0001); this was also true for cocultured U2OS cells between 0 and 100 μM (*p* < 0.0001) and 40 μM and 100 μM (*p* < 0.0004). A similar trend was followed by hMSCs cells, with statistical significant differences in their metabolic activity detected after 72 h between 0 and 100 μM (*p* < 0.0001) and 40 and 100 μM (*p* < 0.006) (Figure 5D). This apparent improved reduction in U2OS cell metabolic activity with the use of PLGA-DOXO NP loaded bone cements validated the decision to use the PLGA-DOXO NP loaded formulation in the following investigations.

#### Osteosarcoma and hMSCs Cytotoxicity

**FIGURE 6:**
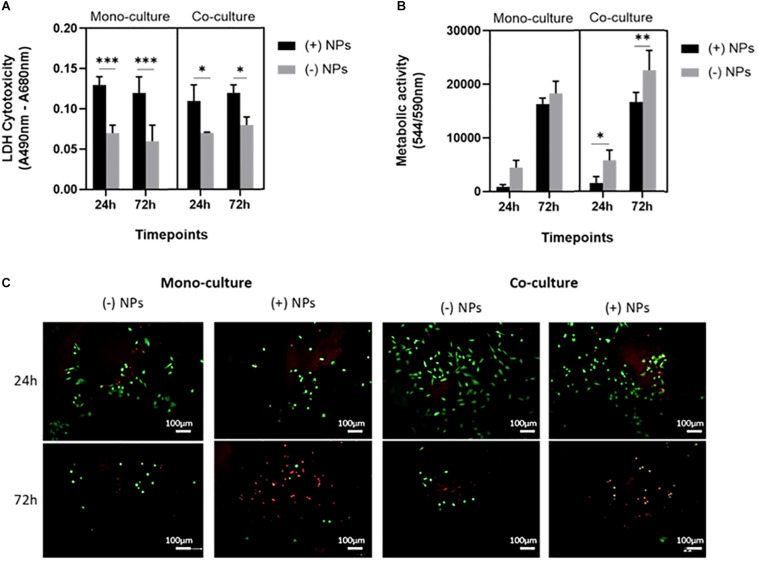
**(A)** Cellular cytotoxicity, **(B)** metabolic activity and **(C)** live dead images (live cells visible in green, dead cells visible in red) of U2OS cells in mono- and co-culture (with hMSCs) grown on bone cements (with 40/60 composition) containing PLGA-DOXO NPs at 40 μM DOXO (+NPs) and without PLGA-DOXO NPs at 0 μM (-NPs, control). Scale bars represent 100 μm. Data presented as mean ± SD (*n* = 3 replicas, *p < 0.05, **p < 0.01 and, ***p < 0.001).

A statistically significant difference was found between mono- and cocultured cells exposed to bone cements (40/60) with and without PLGA-DOXO NPs, at a concentration of 40 μM, after 24 and 72 h, data presented in Figure 6A. Data presented in Figure 6B shows that there was no significant difference in the metabolic activity of U2OS cells monocultured on bone cements (40/60) without PLGA-DOXO NPs (-NP) and with PLGA-DOXO NPs at 40 μM (+NPs) after 24 and 72 h. There was, however, a statistical difference found between the metabolic activity of cocultured U2OS cells on cements fabricated with PLGA-DOXO NPs at 40 μM (+NPs) and without PLGA-DOXO NPs (-NPs) after 24, and 72 h, shown in Figure 6B.

Live and Dead images of U2OS cells in mono- and indirect coculture (with hMSCs) were taken after 24 and 72 h exposure to bone cements (40/60) with PLGA-DOXO NPs (40 μM, +NPs) and without PLGA-DOXO NPs (-NPs), data presented in Figure 6C. After 24 h of incubation, no significant difference between the different culture systems, either in presence or absence of NPs, can be appreciated. However, at 72 h incubation, it is possible to observe a greater amount of live cells (green) on bone cements without PLGA-DOXO NPs (-NPs) if compared to the dead cells (red), in both mono- and cocultures. In contrast, bone cements containing PLGA-DOXO NPs (40 μM, +NPs) showed a greater ratio of dead cells (red) when compared to live cells (green), in both mono- and indirect coculture with hMSCs (Figure 6C).

#### Osteosarcoma Cells Morphology on Bone Cements

**FIGURE 7:**
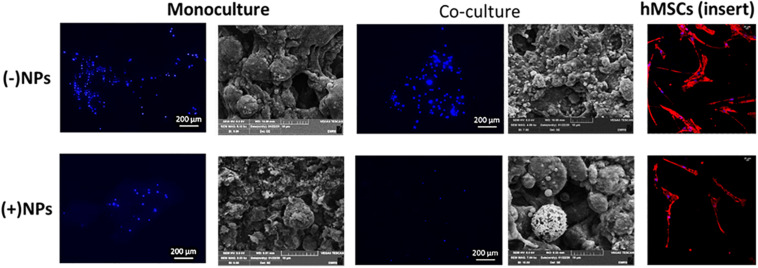
Nucleus staining and cellular-bone cement morphology and interaction of U2OS cells in mono- and indirect co-cultures (with hMSCs) grown on bone cements with a 40/60 composition of CH+G to NHA with and without loaded PLGA-DOXO NPs (40 μM) after 72 h of incubation. Scale bars represent 200, 100, and 20 μm, respectively.

For cements (40/60 composition) without PLGA-DOXO NPs (Figure 7, -NPs), cell nuclei stained in blue (DAPI immunostaining analysis) shows a slight increase of U2OS cells number in monoculture and coculture (135 and 130 nuclei, respectively). Whilst in cements (40/60) containing PLGA-DOXO NPs, fewer nuclei can be observed in monoculture and coculture (27 and 12 nuclei, respectively) indicating a decrease of viable U2OS cells onto these (Figure 7, +NPs). Regarding the hMSCs morphology, nuclei (blue, stained with DAPI) and the cytoskeleton (red, stained with rhodamine-phalloidin), suggests no visible changes in term of morphology independently if seeded in indirect coculture with U2OS cells grown on bone cements with and without PLGA-DOXO NPs (Figure 7). Cytoskeleton of hMSCs appeared typically elongated, spread and attached to the insert’s surface. However, there is an apparent slight reduction in the number of hMSCs present when indirectly cocultured with U2OS cells seeded onto cements (40/60) containing PLGA-DOXO NPs (40 μM), which may indicate a potential cytotoxic effect from released DOXO. SEM analysis of U2OS onto cements (40/60) without PLGA-DOXO NPs showed round-shaped osteosarcoma cells agglomerated after 72 h (Figure 7).

## Discussion

As a major inorganic component of bone, calcium phosphate possesses an excellent biocompatibility in both the dissolved and solid form. Consequently, these are attractive biomaterials for potential hard-tissue applications (Tanzawa et al., 2011). Poor injectability and excessively rapid curing times are common limitations preventing the use of calcium phosphate cements-based into widespread clinical settings (Tariq et al., 2019). Therefore, and increased attention is placed on cement manipulation and appropriate setting times to expand its chemotherapeutic and regenerative capacities. The results obtained in this work showed that cement with a 40/60 composition had the quickest mean setting time (19 ± 0.5 min, shown in Figure 1A). Even though setting-times were improved by decreasing the concentration of biopolymers (CH:G) in bone cement formulations, these must be further improved if intended for clinical applications. In clinical settings, it is desirable to use cements with fast curing time (around 10–15 min), to prevent crumbling of unset cement mixtures when exposed to bodily fluids (Xu et al., 2007).

From results presented in Figure 1B, cements with a 70/30 composition of liquid-to-powder (CH/G:NHA) showed the best injectability properties; however, it performed poorly in mechanical testing after setting (Figure 1C). Broadly, it was observed a trend of increased injectability with the increasing of biopolymers content,. The reduction of the powder-to-liquid ratio has been found to improve CPC injectability due to changes in viscosity, however, it prolongs setting time while jeopardizing the cement mechanical properties (Yousefi, 2019). On this regard, the injectability of the 40/60 composition was found to be 98.6 ± 0.3% effective with minimal cement loss during the process with the highest young’s compressive modulus (97.04 ± 18.04 kPa), offering a good compromise between mechanical properties and easy handling that makes it a suitable injectable cement (Bohner, 2010). In general, the 40/60 composition was found to perform best under increasing levels of force, while mimicking the percentage of CP found in natural bones. Cements classified as CPCs are described as second-generation biomaterials, with compositions closely resembling natural bone (Yousefi, 2019). CPCs with decreased powder components, like NHA, result in decreased mechanical properties (Barralet et al., 2004), seen in the 50/50, 60/40 and 70/30 cements, presented in Figure 1C. NHA is the key inorganic hydration product used in CPCs (Khairoun et al., 1999), in formulated cements (Figure 1D) is found to increase with NHA content evidenced by bands within the range between 3300 and 3500 cm^–1^ (associated to O-H). On this regard, it can be anticipated that the incorporation of NHA may influence CPC resorbability properties (Sakai et al., 2016). In summary and based on shown outcomes on setting time, injectability, mechanical properties; the authors have identified the composition 40/60 ratio of biopolymer (Chitosan and Gelatin):NHA as the best composition to carry forward in further *in vitro* analysis since it demonstrated a good compromise of overall properties as injectable biodegradable material.

The relatively low level of radiopacity associated with CPCs are another reason for their limitation in widespread clinical use (Wu et al., 2018). To overcome this, bismuths, a family of radiopacifiers (Antonijevic et al., 2014), are often used to increase the level of CPC radiopacity (Wu et al., 2018). The ability to track cement placement, and identify any leakage, are both vital to minimize the effects of any complications. Moreover, bismuths have large atomic numbers and for this, they are not toxic and therefore, ideal candidates for the use in CPCs. As reported in Figure 1E, all the compositions analyzed with X-Rays demonstrated that the BS was homogeneously distributed in the polymer, obtaining optically lucent radiopaque cements. The bone cements (40/60 composition) containing BS concentration of 15% w/w showed cements with radiopacity properties as visibly clear as the bone replica controls (as seen when using the phantom). It is anticipated that the radiopaque contrast properties may be improved by a further increase of BS quantity; and consequently, the physical-chemical properties should be also investigated for new formulations.

A lack of degradation can limit bone regeneration (Lodoso-Torrecilla et al., 2019) due to limited space for the formation of new bone tissue (Habraken et al., 2007). Attempts to overcome this have focused on the addition of porogens to CPC compositions (Liao et al., 2011) with PLGA addition proven to be successful in the generation of cement pores (Lanao et al., 2011). This allows for a quicker rate of degradation and improved bone regeneration (Lodoso-Torrecilla et al., 2019). This is a finding reflected in the data presented in this work, with increased pore sizes and decreased mass of cements containing PLGA NPs visible after 14 days (Figure 1F). From this result, it can be anticipated that over a prolonged period, cements of a 40/60 composition containing loaded PLGA-DOXO NPs would degrade further following this time, allowing for bone tissue regeneration. However, increase porosity and mass loss would also point toward a potential decrease in the mechanical properties, which could also impact regeneration rates. Also, the addition of DOXO, whether free or loaded in PLGA-DOXO NPs, affected the porosity of cements, as seen in Supplementary Figure S3A. Although, potential reasons can be foreseen, including (1) the presence of NH_2_ groups in DOXO molecule that could potentially react with unreacted NH_2_ groups (either from CH or G), if exists an excess of Genipin; or (2) a potential interaction of DOXO and loaded-DOXO NPs with water or other components, leading to an increased hydration capability and consequent changes in bone cements porosity, after freeze-drying. However, any of these theories should be further investigated and proven. As reported elsewhere, the porosity of CPCs can be affected via the use and interaction with different materials, including polymers (Shariff et al., 2016), calcium carbonates (Espanol et al., 2009), foaming agents (Ginebra et al., 2007), etc.

PLGA-DOXO NPs obtained via the nano-precipitation method showed a spherical morphology and a narrow, mono-modal size distribution. Small size nanoparticles, at ∼150 nm (Figure 3B), were obtained with a DOXO EE of 32.7 ± 0.6%. Our data for EE is in agreement with previously published work. For instance, Mattu et al. reported an EE of DOXO ranging from 22 to 45% inside block-copolymer nanoparticles, with EE increasing with increasing amount of hydrophilic blocks in the co-polymer composition (Mattu et al., 2018). Similarly, Dessy and collaborators reported EE of hydrophilic DOXO to be in the range 22–26%(Dessy et al., 2012). Doxorubicin is a water soluble drug, which may easily diffuse toward the water phase during the nanoprecipitation process, explaining the relatively low values of EE. The data presented in Figure 3C show that PLGA-DOXO NPs are evenly distributed throughout the bone cement and preserve their spherical morphology, indicating that the cement preparation process did not affect the integrity of the NPs (Ferreira et al., 2014; Gentile et al., 2016).

A minimum of 24 h is required for endocytosis of DOXO before U2OS spheroid undergo cellular arrest (Baek et al., 2016). However, following tumor removal minimal cancerous cells remain, therefore a 2D U2OS model may prove more beneficial in understanding therapeutic bone cement potential. The U2OS cultures in 2D showed a significantly lower IC50 of DOXO, a trend reflected in hMSCs. This implies the concentration of DOXO administered locally via bone cement, would be reduced compared to pre-adjuvant treatments. This suggests chemotherapeutic resistant mechanisms present in spheroids do not occur in 2D cultures. The lack of architectural complexity in 2D cultures allows for easier drug penetration compared to 3D cultures (Imamura et al., 2015). While in the undifferentiated state, hMSCs have increased resistance to DOXO compared to their differentiated counterparts (Kozhukharova et al., 2018). These chemoprotective properties, under the correct conditions could be passed onto OS cells because of MSC education (Sun et al., 2014), thus further investigations into 2D co-cultures containing both U2OS and hMSCs is essential.

Data presented in Figure 6A showed a significant difference in cytotoxicity of monocultured U2OS and cocultures U2OS with hMSCs cells grown on cements in presence and absence PLGA-DOXO NPs after 24 and 72 h. More cells survived on cements without NPs, in both cases. Furthermore, immunostaining and SEM analysis, presented in Figure 7 confirmed this tendency, with less cells found in the NPs loaded samples. Taken together, these results suggested that adequate levels of DOXO have been released from loaded NPs, thus resulting in increased levels of cytotoxicity, inhibiting U2OS growth and inducing their apoptosis. The presence of hMSCs allows for a more accurate representation *in vitro* model mimicking the complexity of the OS tumor microenvironment. Data presented in Figure 5 shows significant differences between U2OS cells cocultured with hMSCs on varying concentrations of bone cements with PLGA-DOXO NPs, after 24 and 72 h. However, following 72 h culturing with cements containing free DOXO, there was no significant difference between U2OS cells in mono- or coculture with hMSCs on varying concentrations of bone cement. As reported by Sun et al. (2014), when close to the tumor site, hMSCs undergo the “educational” process via the acquisition of TAF phenotype, promoting OS cells proliferation and tumor growth, suppressing the inhibition effect of DOXO on them. These findings demonstrate that the presence of hMSCs is sufficient to suppress even a high (100 μM) dosage of DOXO in free drug cements, explaining why no differences were found between the different drug concentrations in our work. Resistance during the undifferentiated stage is vital in ensuring that the tumor microenvironment is maintained, as U2OS cells produce increased levels of transforming growth factor-β (TGF-β) preventing hMSC differentiation into osteoblasts (Tu et al., 2014). Maintenance of an undifferentiated state in hMSCs is thought to increase the production of pro-tumor cytokines (Tu et al., 2014), explaining why no significant difference was seen between U2OS cells cocultured with hMSCs on increasing concentrations of free DOXO bone cements.

## Conclusion

In conclusion, bone cements containing PLGA-DOXO NP have better-controlled DOXO release over 7 days compared to bone cements containing free DOXO, suggesting a better approach for modulating drug delivery at tumor sites. To prolong the release of DOXO from these cements, the combination of free DOXO and PLGA-DOXO NPs should be considered in future works. The presence of hMSCs offer a degree of DOXO resistance in U2OS cells cultured on PLGA-DOXO NP bone cements. This multidirectional attack of OS cells could support apoptosis of residual cancer cells, as well as minimize the effects of hMSCs that display pro-tumor characteristics. Still, the effectiveness of this approach to treat osteosarcoma needs to be studied in animal models. These findings contribute to pre-existing knowledge in the field of bioengineering involving bone reconstruction and NP technology for the localized controlled delivery of drugs. By combining these two disciplines there is an exciting potential to create a positive impact in cancer treatment of osteosarcoma, offering a less demanding form of adjuvant chemotherapy to future patients.

## Data Availability Statement

Data supporting this publication is openly available under an “Open Data Commons Open Database License.” Additional metadata are available at: doi: 10.25405/data.ncl.12047292. Please contact Newcastle Research Data Service at rdm@ncl.ac.uk for access instructions.

## Author Contributions

RD, AF, PG, CM, and KR contributed to the conception and design of the study. RD, AF, JB, and AS collected the data. RD, AF, and JB performed the statistical analysis. RD, AS, and JB wrote the first draft of the manuscript. All authors contributed to the article and approved the submitted version.

## Conflict of Interest

The authors declare that the research was conducted in the absence of any commercial or financial relationships that could be construed as a potential conflict of interest. The handling Editor LR declared past co-authorship with several of the authors, PG and AF.

## Supplementary Material

FIGURE S1 **(A)** Mean contrast of bone cements bone cements with CH+G to NHA composition of 40/60, 50/50, 60/40, and 70/30 imaged at 40 kV/11 mAs. **(B)** Mean contrast of bone cements bone cements with CH+G to NHA composition of 40/60, 50/50, 60/40, and 70/30 imaged at 40 kV/5.6 mAs. **(C)** X-ray images of bone cements with 40/60, 50/50, 60/40, and 70/30 compositions taken at t 40 kV/5.6 mAs. **(D)** X-ray images of bone cements with 40/60, 50/50, 60/40, and 70/30 compositions taken at 40 kV/5.6 mAs. (*n* = 3 replicas ^∗^*p* < 0.05, ^∗∗^*p* < 0.005).

FIGURE S2 FTIR-ATR spectra, of bone cements with **(A)** 40/60, **(B)** 50/50, **(C)** 60/40, and **(D)** 70/30 compositions of CH+G to NHA. **(E)** Mean changes in pH of bone cements (40/60) containing no DOXO (0 μM), 40 μM Free DOXO and 100 μM PLGA-DOXO NPs over 3 days incubation in PBS at 37°C (*n* = 3 replicas).

FIGURE S3 **(A)** SEM images of 0 μM, 40 μM Free DOXO and 100 μM PLGA-DOXO NP bone cements (40/60 composition). Scale bars represent 1 mm. **(B)** Mean Pore Area of bone cements (40/60) in absence of DOXO (0 μM), Free DOXO (dissolved DOXO at 40 μM) and PLGA-DOXO NPs (at concentration 100 μM). Data presented as mean ± standard deviation, (*n* = 3 replicates) ^****^*p* < 0.001.

FIGURE S4 Mean cell viability of U2OS cells in monoculture, U2OS cells in coculture and hMSCs in coculture grown on bone cements with a 40/60 CH+G to NHA composition on cements without Free DOXO (0 μM) and with Free DOXO (40 μM and 100 μM) after **(A)** 24 and **(B)** 72 h. Mean cell viability of U2OS cells in monoculture, U2OS cells in coculture and hMSCs in coculture grown on bone cements with a 40/60 CH+G to NHA composition on cements without PLGA-DOXO NPs (0 μM) and with PLGA-DOXO NPs (40 μM and 100 μM) after **(C)** 24 and **(D)** 72 h (*n* = 3 replicas, ^∗^*p* < 0.05, ^∗∗^*p* < 0.005, ^∗∗∗^*p* < 0.001).
