# Supplementary figures and images for: Development of Natural-Based Bone Cement for a Controlled Doxorubicin-Drug Release
Source: Front Bioeng Biotechnol. 2020 Jul 9;8:754. doi: 10.3389/fbioe.2020.00754 (PMC7363953; doi:10.3389/fbioe.2020.00754)

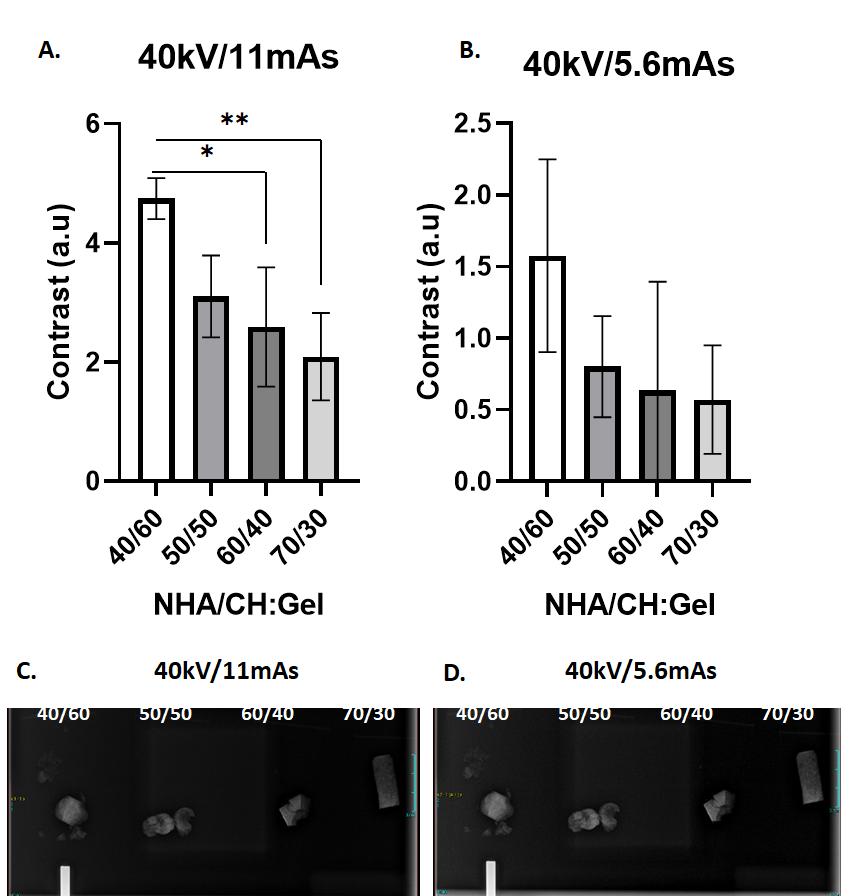

Supplement: FIGURE S1 — (A) Mean contrast of bone cements bone cements with CH+G to NHA composition of 40/60, 50/50, 60/40, and 70/30 imaged at 40 kV/11 mAs. (B) Mean contrast of bone cements bone cements with CH+G to NHA composition of 40/60, 50/50, 60/40, and 70/30 imaged at 40 kV/5.6 mAs. (C) X-ray images of bone cements with 40/60, 50/50, 60/40, and 70/30 compositions taken at t 40 kV/5.6 mAs. (D) X-ray images of bone cements with 40/60, 50/50, 60/40, and 70/30 compositions taken at 40 kV/5.6 mAs. (n = 3 replicas ∗p < 0.05, ∗∗p < 0.005). [file Image_1.TIF]

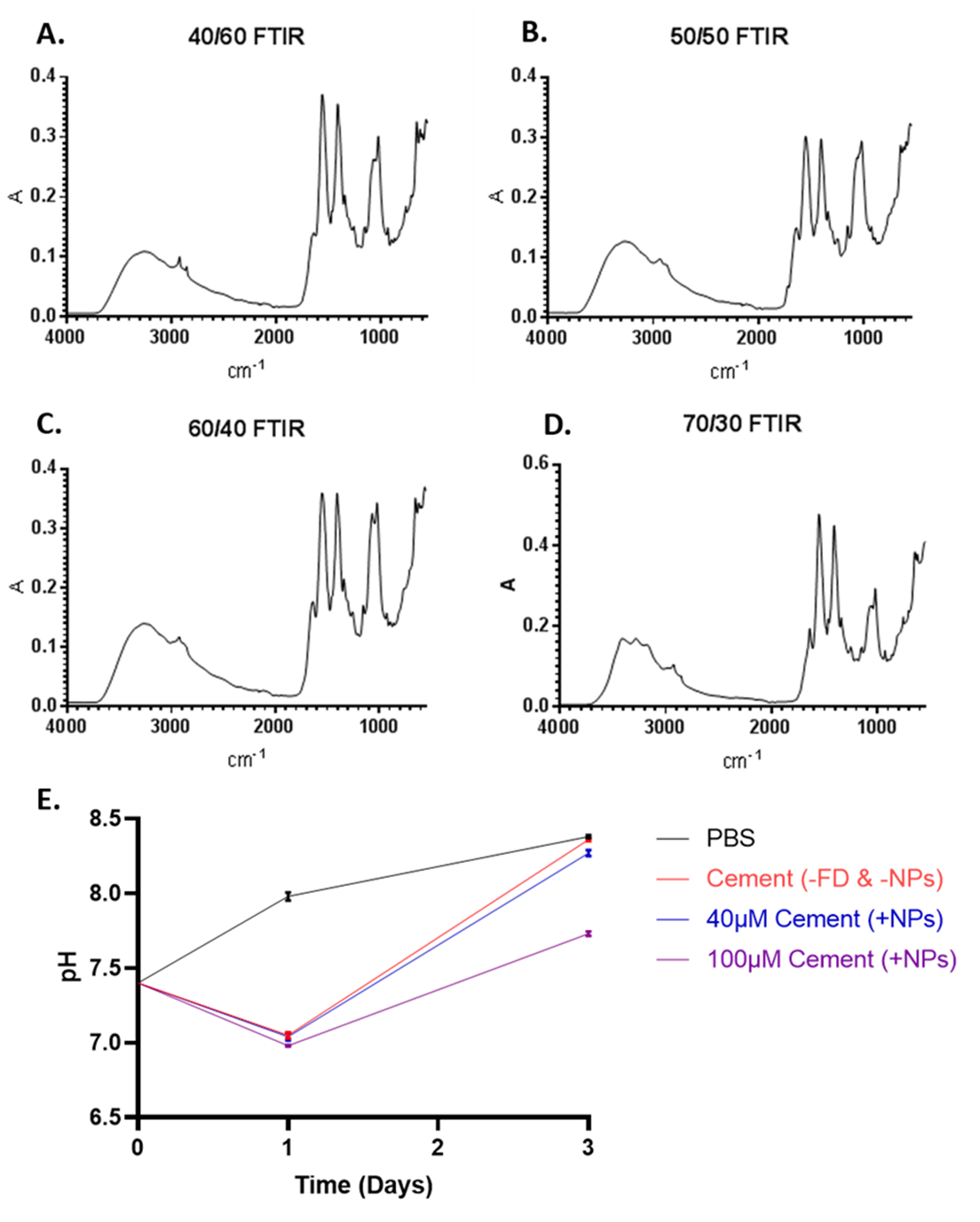

Supplement: FIGURE S2 — FTIR-ATR spectra, of bone cements with (A) 40/60, (B) 50/50, (C) 60/40, and (D) 70/30 compositions of CH+G to NHA. (E) Mean changes in pH of bone cements (40/60) containing no DOXO (0 μM), 40 μM Free DOXO and 100 μM PLGA-DOXO NPs over 3 days incubation in PBS at 37°C (n = 3 replicas). [file Image_2.TIF]

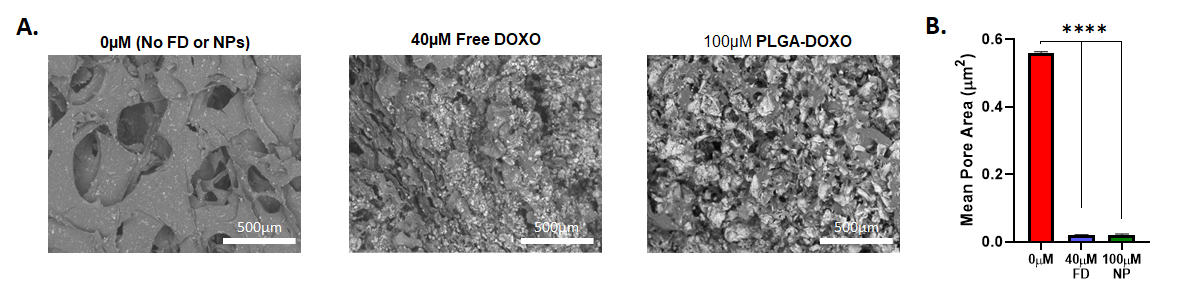

Supplement: FIGURE S3 — (A) SEM images of 0 μM, 40 μM Free DOXO and 100 μM PLGA-DOXO NP bone cements (40/60 composition). Scale bars represent 1 mm. (B) Mean Pore Area of bone cements (40/60) in absence of DOXO (0 μM), Free DOXO (dissolved DOXO at 40 μM) and PLGA-DOXO NPs (at concentration 100 μM). Data presented as mean ± standard deviation, (n = 3 replicates) ****p < 0.001. [file Image_3.TIF]

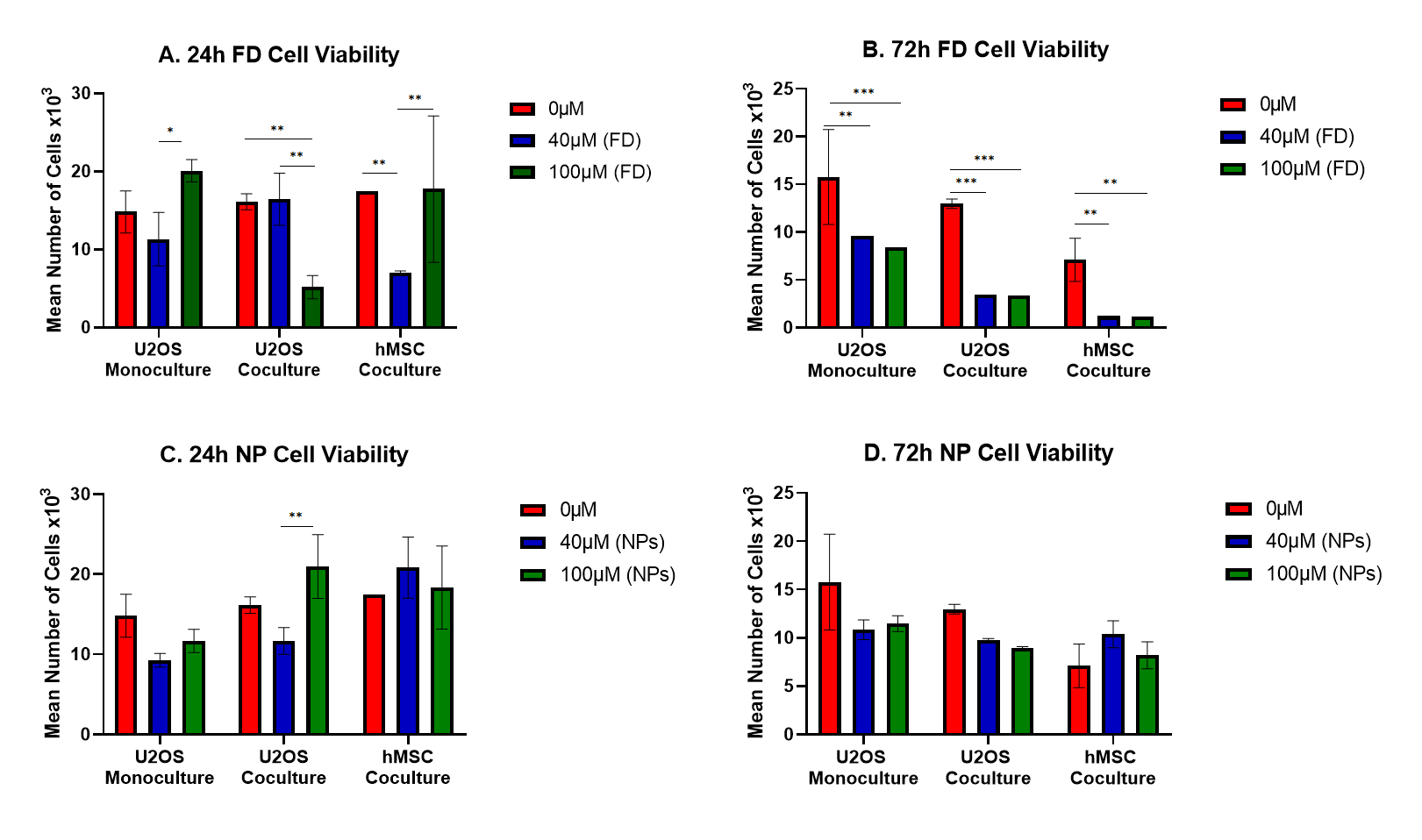

Supplement: FIGURE S4 — Mean cell viability of U2OS cells in monoculture, U2OS cells in coculture and hMSCs in coculture grown on bone cements with a 40/60 CH+G to NHA composition on cements without Free DOXO (0 μM) and with Free DOXO (40 μM and 100 μM) after (A) 24 and (B) 72 h. Mean cell viability of U2OS cells in monoculture, U2OS cells in coculture and hMSCs in coculture grown on bone cements with a 40/60 CH+G to NHA composition on cements without PLGA-DOXO NPs (0 μM) and with PLGA-DOXO NPs (40 μM and 100 μM) after (C) 24 and (D) 72 h (n = 3 replicas, ∗p < 0.05, ∗∗p < 0.005, ∗∗∗p < 0.001). [file Image_4.TIF]
